# Education of future paramedics - variation in level of experience when entering paramedic training. A quantitative pilot study

**DOI:** 10.1186/1757-7241-23-S1-A1

**Published:** 2015-07-16

**Authors:** Rasmus Malmberg-Lyngby, Martin Betzer

**Affiliations:** 1 Pre-hospital system, Region East, Denmark; 2 Pre-hospital system, Region Copenhagen, Denmark

## Background

After three years of employment an ambulance technician (AT) can start paramedic education. The period of three years is dictated by law and does not include any benchmarking of individual skills or number of cases handled. The aim of this study is to investigate if there are major differences in levels of experience for ATs, focusing on numbers of patients handled, drug administrations, use of skills, and patient categories as variables.

## Methods

We obtained data on the number of patients treated annually by the ambulance service from "Tender for Ambulance Service in the Copenhagen Region" (Region Hovedstaden). Highest and lowest patient flows were identified and compared. Data on drug administrations, skills, and patient categories were obtained from an AT questionnaire survey. Highest and lowest frequencies were identified and compared. All data obtained was categorized following the same geographical sub areas as in the "Tender for Ambulance Service in the Copenhagen Region".

## Results

Patient flow data shows a significant difference between the 6 sub areas in the region. Areas with the lowest patient flow allow for the average AT to obtain 565 patient contacts per year, while ATs in a high flow area could obtain 1,200 patient contacts - a difference of 112%. Patient flow data is confirmed by the questionnaire survey that also reveals that the number of calls dispatched to units vary, which allows for a larger difference in patient flow if measured on the individual AT and not the average of an area. The questionnaire survey also reveals that administrations of drugs, use of skills, and patient categories for the individual AT have significant variations. Differences in drug administrations have shown to be in a ratio of up to 8:1, skills 49:1, and patient categories 18:1.

## Conclusion

The study showed significant differences in patient contacts, drug administrations, and use of skills. The study revealed that a three-year-period as the only benchmark of experience is an unreliable method of measuring or determining the individual experience in student paramedics. Timeframe based benchmarks will not give a uniform level of experience and have shown to be an unreliable method to measure pre-hospital experience.

## Acknowledgements

Mikkel Brabrand, Peter Hallas, Lars Folkestad, Christian B Laursen

